# A80 CANADIAN NEUROGASTROENTEROLOGY NETWORK (CNN) SURVEY ON PH/MOTILITY TESTING IN CANADA

**DOI:** 10.1093/jcag/gwab049.079

**Published:** 2022-02-21

**Authors:** D E Reed, M J Beyak, D Rodrigues, S Vanner, W G Paterson

**Affiliations:** Queen’s University, Kingston, ON, Canada

## Abstract

**Background:**

Anecdotal reports suggest that access to pH/motility testing is problematic in Canada, but to date there is little data documenting this.

**Aims:**

To assess the volume and accessibility of motility lab testing in Canada.

**Methods:**

The CNN developed a questionnaire directed at the scope, volume and accessibility of pH/motility testing in Canadian labs. Fifty-three labs were identified using lists provided by companies that supply pH/motility recording equipment in Canada. Of these, 12 labs were excluded (10 had incorrect or absent contact information, 1 had recently closed and 1 had just opened). Questionnaires were sent in early 2020 to the remaining 41 labs, and respondents were asked to use data from their last fiscal year pre-pandemic.

**Results:**

26 completed questionnaires were returned (i.e., 63% response rate, but representing ~ 51% of active labs): 23 adult units (7 community, 15 academic and 1 private) and 3 academic pediatric units. Of the adult units, 6 performed studies in children <12 yrs old. All 3 pediatric units provided both esophageal and anorectal high-resolution manometry (HRM) and pH/Impedance recording, with wait times of < 3 months. All 23 adult labs provided esophageal HRM, but just 50% performed anorectal manometry and only 3 anorectal manometry with biofeedback. Ambulatory pH/Impedance was performed in all but 1 adult unit. 15 of 23 adult centres reported access to colon transit studies and only one performed colonic manometry. No units performed antroduodenal manometry. Five units offered Bravo wireless pH recording and 4 performed ENDOFLIP. In adult units, the median number of procedures per year were as follows: esophageal HRMs - 278 (range: 50–1140); pH/impedance - 225 (range: 40–634); anorectal manometry - 90 (range: 10–450). Corresponding median wait times in months were as follows: esophageal HRM - 4 (range: 0.5–14); pH/Impedance - 4.5 (range: 0.5–14); anorectal manometry - 4.6 (range: 2–9). Only 6 of the 23 adult units met recommended wait time targets of <2 months. Testing was performed by a nurse in ~ 80% of centres, while testing was done by technicians in 2 units and physicians in 3 units. 8 units accepted referrals from primary care physicians, whereas the remainder only accepted specialist referrals. 50% screened referrals for appropriateness and restricted access accordingly.

**Conclusions:**

The scope of motility and pH testing across Canada is variable, with lower GI testing lacking in many regions. Wait times vary significantly across labs and the majority of centres exceed recommended limits of 2 months. The reasons underlying the identified limitations to pH/motility testing access warrant further study.

**Funding Agencies:**

None

